# Effects of Soy Isoflavones on Glycemic Control and Lipid Profile in Patients with Type 2 Diabetes: A Systematic Review and Meta-Analysis of Randomized Controlled Trials

**DOI:** 10.3390/nu13061886

**Published:** 2021-05-31

**Authors:** Agnieszka Barańska, Agata Błaszczuk, Małgorzata Polz-Dacewicz, Wiesław Kanadys, Maria Malm, Mariola Janiszewska, Marian Jędrych

**Affiliations:** 1 Department of Medical Informatics and Statistics with E-Learning Lab, Medical University of Lublin, 20-090 Lublin, Poland; maria.malm@umlub.pl (M.M.); mariola.janiszewska@umlub.pl (M.J.); marian.jedrych@umlub.pl (M.J.); 2 Department of Virology with SARS Laboratory, Medical University of Lublin, 20-093 Lublin, Poland; agata.blaszczuk@umlub.pl (A.B.); malgorzata.polz.dacewicz@umlub.pl (M.P.-D.); 3 Specialistic Medical Center Czechow, 20-848 Lublin, Poland; wieslaw.kanadys@wp.pl

**Keywords:** T2DM, soy isoflavones, lipid profile, total cholesterol, HDL-C, LDL-C, triacylglycerol, glycemic control, HbA1c, HOMA-IR

## Abstract

The aim of the report was to investigate the impact of soy protein and isoflavones on glucose homeostasis and lipid profile in type 2 diabetes. The studies used in this report were identified by searching through the MEDLINE and EMBASE databases (up to 2020). Meta-regression and subgroup analyses were performed to explore the influence of covariates on net glycemic control and lipid changes. Weighted mean differences and 95% confidence intervals (CI) were calculated by using random-effect models. Changes in the lipid profile showed statistically significant decreases in total cholesterol and LDL-C concentrations: ‒0.21 mmol/L; 95% CI, ‒0.33 to ‒0.09; *p* = 0.0008 and ‒0.20 mmol/L; 95% CI, ‒0.28 to ‒0.12; *p* < 0.0001, respectively, as well as in HDL-C (−0.02 mmol/L; 95% CI, −0.05 to 0.01; *p* = 0.2008 and triacylglycerols (−0.19 mmol/L; 95% CI, −0.48 to 0.09; *p* = 0.1884). At the same time, a meta-analysis of the included studies revealed statistically insignificant reduction in fasting glucose, insulin, HbA1c, and HOMA-IR (changes in glucose metabolism) after consumption of soy isoflavones. The observed ability of both extracted isoflavone and soy protein with isoflavones to modulate the lipid profile suggests benefits in preventing cardiovascular events in diabetic subjects. Further multicenter studies based on larger and longer duration studies are necessary to determine their beneficial effect on glucose and lipid metabolism.

## 1. Introduction

Diabetes mellitus has been widely recognized to be a fundamental and leading cause of major health issues, such as cardiovascular disease. The world prevalence of diabetes among adults (aged 20–79 years) amounted to 285 million adults in 2010, and will increase to 439 million adults by 2030 [1]. In the United States, in 2018, 34.2 million people were thought to be diabetic (10.5% of the U.S. population), including 26.9 million people (26.8 million adults) confirmed and 7.3 million unconfirmed (21.4%) [2]. Obesity and diabetes are major causes of morbidity and mortality in the United States [2]. 

T2DM is characterized by elevated fasting plasma glucose (FPG), insulin resistance and relative lack of insulin [3,4]. A variety of metabolic disorders, such as obesity, hypertension and dyslipidemia very often coexist with diabetes [5,6]. Lifestyle factors, particularly those associated with obesity, and a rapid increase in the intake of fat, notably saturated fatty acids, as well as a decrease in physical activity contribute to developing T2DM [7,8].

Improvements in glycemic control have been demonstrated in adults with T2DM through a combination of pharmaceuticals and lifestyle changes, and with lifestyle changes alone [9,10]. Lifestyle factors such as diet and physical activity can be individually modified. It is important to choose a diet in relation to the quality of nutrients, including carbohydrates, protein, fats, minerals and vitamins, and to establish its health benefits [11,12]. A number of studies on animal models [13,14,15] and intervention studies in humans [16,17,18,19,20] have shown that soy protein with isoflavones can improve the parameters of glycemic control and lipid homeostasis. Recently, several new studies on this topic have appeared [21,22,23]. 

This systematic review and meta-analysis was undertaken to investigate the influence of soy isoflavones on glucose metabolism, including fasting blood glucose (FBG), fasting insulin (FI), glycosylated hemoglobin A1 level (HbA1c) and peripheral insulin resistance (homeostasis model assessment of insulin resistance: HOMA-IR), as compared with healthy subjects, in patients with T2DM. A secondary aim of the study was to evaluate the influence of soy isoflavones on lipid metabolism. 

## 2. Materials and Methods

### 2.1. Search Strategy and Study Selection

The study was conducted based on the PRISMA guidelines, and utilized the MEDLINE (PubMed) and EMBASE electronic database websites (up to March 2020) [24]. The following search words were used in various combinations to identify relevant studies: diabetes mellitus, T2DM, type 2 diabetes mellitus, soy protein, soy isoflavones, lipids, lipid profile, cholesterol, glucose metabolism, glucose control, and randomized controlled trials. Inclusion criteria were: randomized controlled trials; parallel-group design, or crossover design that contained data for the first period; studies that provided sufficient information on the values of FG, FI, HbA1c, and HOMA-IR, as well as total cholesterol (TC), LDL-cholesterol (LDL-C), HDL-cholesterol (HDL-C), and triacylglycerols (TAG) before and after administration of isoflavones; studies that a daily dose of soy isoflavones; and involved a comparison with a placebo or with a no-intervention group. The exclusion criteria were as follows: no control group in the study, lack of sufficient information, results were reported as graphics or percent changes, and as duplicated reports.

### 2.2. Data Extraction

The following data was extracted from each of the included studies: first author’s name, year of publication, country of origin, study design; follow-up period, number of participants in the intervention and control groups; characteristics of the studied populations (age (range), menopausal status (years since menopause), body mass index), daily dose of soy isoflavones, type of control group, and initial and final mean values with corresponding standard deviations (SD) of the above-mentioned components of the lipid metabolism and glycemic profile, for each comparison group. When different units were given in the research (conventional units or System International of Units [SI]), the following conversion factors were used to unify them: to convert cholesterol to mmol/L, multiply by 0.02586; to convert triglycerides to mmol/L, multiply by 0.01113; to convert insulin to pmol/L, multiply by 6; to convert glucose to mmol/L, multiply by 0.05551; and to convert HbA1c to %, multiply by 0.0915 + 2.15. To avoid duplication of data in trials with multiple time points, only the results from the shortest follow-up were taken into account. In the case of trials with more than one active group compared to one control group, all results were taken into account.

### 2.3. Quality Assessment and Bias Risk of the Trials

The quality of trials was evaluated using the Cochrane Collaboration’s tool [25]. This consists of seven items that have a potential biasing influence on the estimates of intervention effectiveness in randomized studies. Included are: selection bias (random sequence generation and allocation concealment), performance bias (blinding of participants and personnel), detection bias (blinding of outcome assessment), attrition bias (incomplete outcome data), reporting bias (selective reporting), and other sources of bias. The risks of bias in RCTs are designated in the review as ‘high risk’, ‘unclear’, or ‘low risk’ [25]. To explain the possible presence of bias publications, Begg’s rank correlation test (Kendall Tau) and Egger’s weighted regression test were applied [26,27]. 

### 2.4. Statistical Analysis and Meta-Analysis

Treatment effect of each comparison group was defined as the mean difference (MD) (final value minus baseline value) from corresponding SD of change in individual components of lipid metabolism or glycemic profile for subjects ingesting soy isoflavones or control. When the standard error of the mean (SEM) was employed, the conversion to SD was made according to the formula: SD = SEM × √N. If a 95% confidence interval (95% CI) was applied, SD conversion was: SD = sqrt (N) × (upper bound–lower bound)/(2u) (equal to 3.96). The missing SD of MD were imputed using the formula: SD = sqrt ((SD ”initial”)^2^ + (SD ”final”)^2^ − (SD ”initial” × SD ”final”) × 2R), where R is the correlation coefficient; we took an R value = 0.50 according to the suggestion of Follmann et al. [28,29]. Summary outcomes measures were presented as mean differences between the intervention and control groups. A random-effects model was used to calculate weighted-mean difference (WMD) and 95% confidence interval (CI) for each comparison, and the combined overall effect (*p* < 0.05 was considered statistically significant), according to DerSimonian and Laird [30]. For heterogeneity evaluation, Cochrane Q and I^2^ statistic were employed. The I^2^ test allowed assessing whether the variance across studies was correct and not due to sampling errors. Percentage of total variation indicates the degree of heterogeneity; I^2^ values of ≤25% were considered low, >25% as moderate, and ≥75% as high heterogeneity [31]. Multivariate meta-regression was also applied. Since this is a multivariate regression, its results differ from the subgroup analysis.

### 2.5. Subgroup Analysis

Additional subgroup analyzes were performed in order to detect sources of heterogeneity according to the following covariate variables: design of studies (parallel vs. crossover), participants age (≤60 y vs. >60 y), follow-up period (≤8 w vs. >8 w), BMI (<30 kg/m2 vs. ≥30 kg/m^2^), duration of diabetes (<5 y vs. ≥5 y) and isoflavones dose per day (<80 mg vs. ≥80 mg) [32]. Furthermore, meta-regression was undertaken to investigate whether there were any strong predictors of lipid and glycemic changes [33]. 

## 3. Results

**Figure 1:**
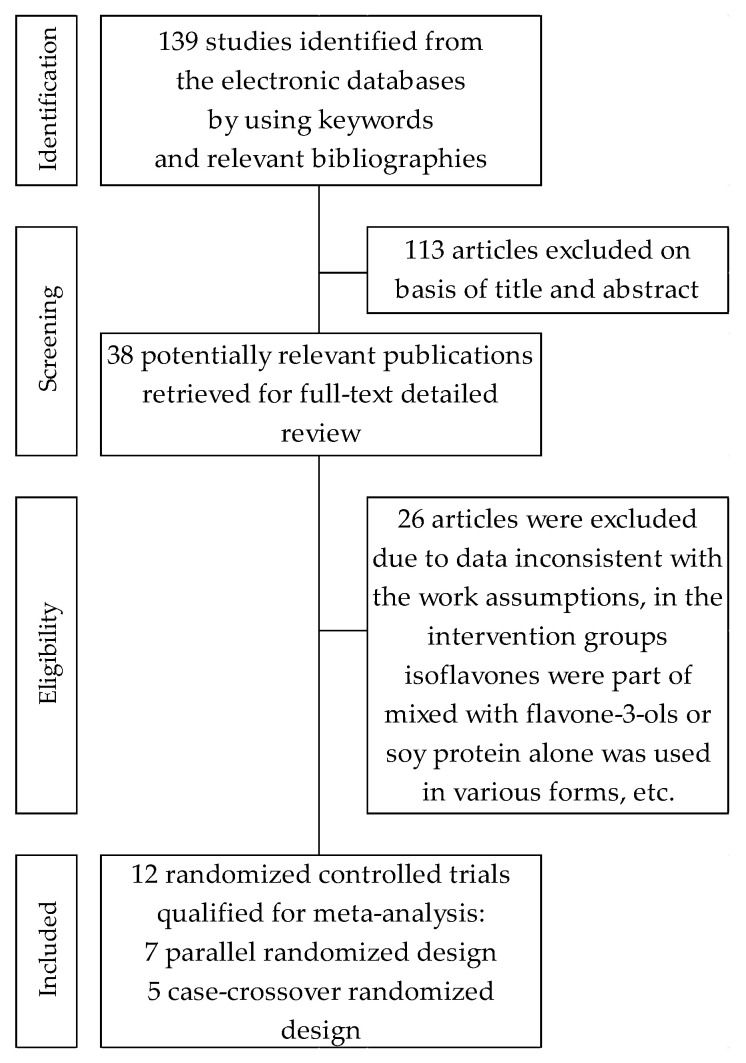
Flowchart of the selection procedure for studies included in the current review and meta-analysis.

Our search yielded 139 citations for double screening of abstracts, of which 38 were identified for full-text analysis. Of these, 12 randomized controlled trials were finally included. A detailed review of selection procedures is shown in Figure 1. Of the total of 12 articles included in this meta-analysis: (a) nine reported on glucose metabolism [21,22,34,35,36,37,38,39,40], including eight that concerned FBG [21,22,34,35,36,37,39,40], seven dealt with FI [21,22,34,35,37,39,40], five were about HbA1c [21,22,38,39,40] and five were homeostasis model assessments of HOMA-IR [21,22,34,35,37,39]; and (b) nine reported lipid profiles [21,22,23,36,37,39,40,41,42]. Of the selected trials, five studies were of parallel randomized design [21,22,23,34,36], and seven studies used cross-over randomized design [35,37,38,39,40,41,42]. Here, the crossover researches conducted by Gobert et al. [35] and Pipe et al. [41] are based on identical characteristics, but differed in the parameters assessed in the analysis: glucose or lipid profile, respectively.

### 3.1. Characteristics of Included Trials

The characteristics of selected studies are listed in Table 1. In total, the 12 trials involved 662 participants (56.2% women and 43.8% men) in mean age at baseline of 59.7 ± 10.3 (10 trials reporting). In trials reporting gender distribution, four trials consisted entirely of women [23,34,37,39], two trials consisted entirely of men [22,38], and six trials consisted of men and women [21,35,36,40,41,42] Of trials that reported T2DM, five studies involved subjects with obesity [21,22,37,39,40], of which one study was carried out on diabetes complications that pertained to subclinical hypogonadism [22]; three studies dealt with diabetic nephropathy [36,38,42]; and four studies involved subjects without any complications [23,34,35,41]. The duration of the trials in most studies ranged from 6 weeks to 12 weeks. Only two studies examined the effect of longer-term administration of isoflavones [34,36]. The analysis was based on 12 studies, of which nine studies employed isolated soy protein containing isoflavones [21,22,35,36,38,39,40,41,42], one study dealt with two active groups using supplements containing, respectively, milk protein or soy protein enriched in isoflavones [34], and two studies were about the administration of tablets containing extracted isoflavones [23,37]. Compared with the intervention group studies, the majority of the studies used comparator controls: soy protein without isoflavones [21,22], milk protein [34,35,41], animal protein [36,42], microcrystalline cellulose [39], and casein alone [38] or mixed with cellulose [40]—all in the form of powder. Two studies compared isoflavones alone with placebo-in the form of capsules containing starch [23] or microcrystalline cellulose in pills [37]. In five researches, the T2DM participants additionally had intake of various types of antidiabetic drugs (insulin, oral hypoglycemic drugs) [21,22,36,38,40], while in five other researches, T2DM patients did not receive any other drugs [34,35,37,39,41]. No data were available in two studies [23,42]. The included studies were characterized by wide administration of isoflavones: ranging from 32 mg to 435 mg per day. In seven studies, isoflavones were expressed in aglycone units [23,34,35,37,38,40,41], one used glycosides [39] and the form of isoflavones could not be determined in four studies [21,22,36,42].

### 3.2. Assessment of the Methodological Quality of Trials

**Figure 2:**
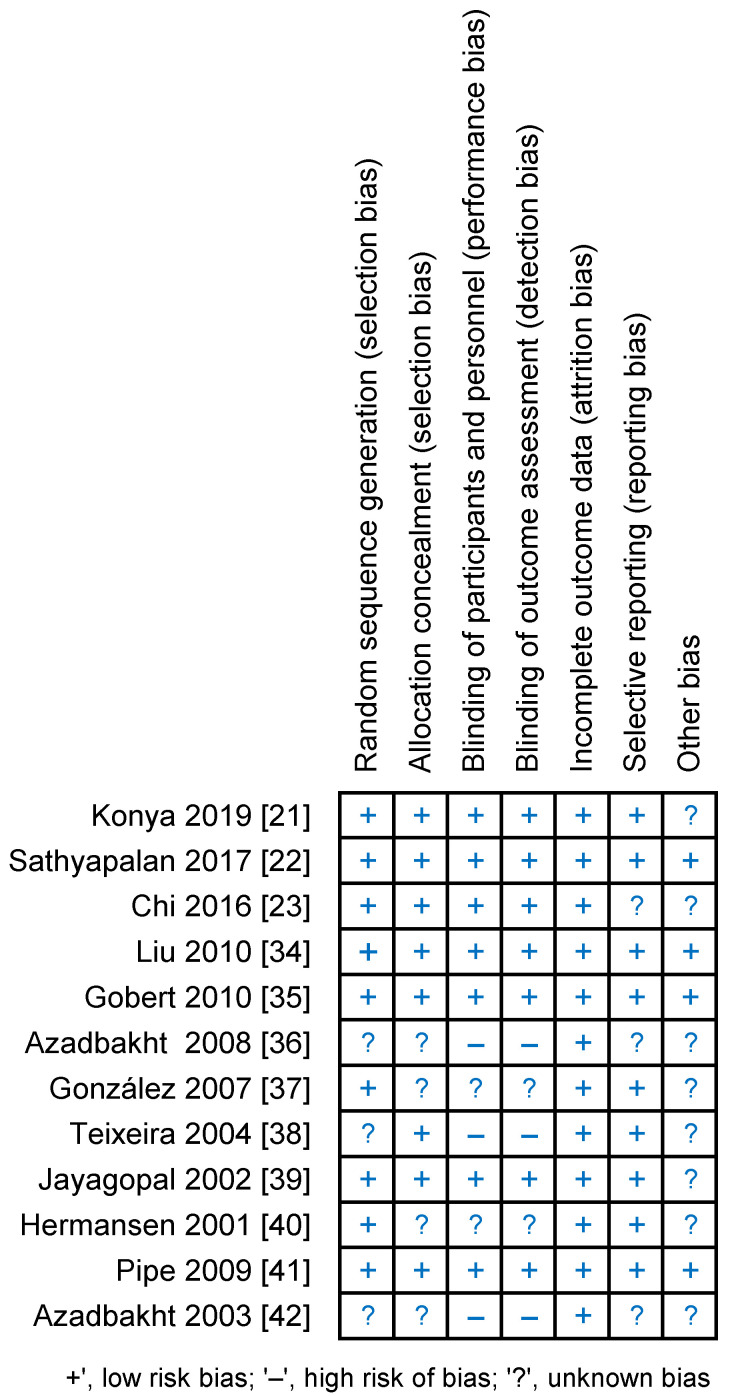
Risk of bias summary for each study-as assessed by the authors.

**Figure 3:**
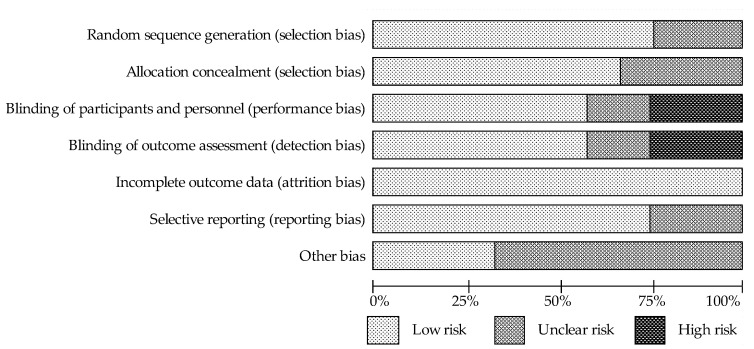
The assessment of risk of bias for each items; data are shown as percentage for studies.

Details of the risk of bias assessment are shown in Figure 2 and Figure 3 . It should be noted that the studies showed the highest risk of bias with regard to blinding. In the categories “blinding of participants and investigators” and ‘‘blinding assessment of the outcomes”, seven (58%) trials were assessed as low risk, two (17%) trials were assessed as unclear risk, which was related to the lack of accurate information on blinding, and three (25%) trials were at high risk due to lack of blinding. Although two of the aforementioned studies have been classified as high risk of bias, it has been proposed that the lack of blinding had little effect on the results [38,42]. In contrast, the low risk categories in 67–75% of the studies were “random sequence generation”, “allocation concealment” and “selective reporting”, and the remaining studies were judged to be of unclear risk due to insufficient information on the methods used by researchers to randomly assigning participants to groups and in reporting all predefined results. In terms of the random sequence generation, 100% of the studies showed low risk of bias.

### 3.3. The Effect of Soy Isoflavones on Metabolism Glucose in Patients with Type 2 Diabetes

**Figure 4:**
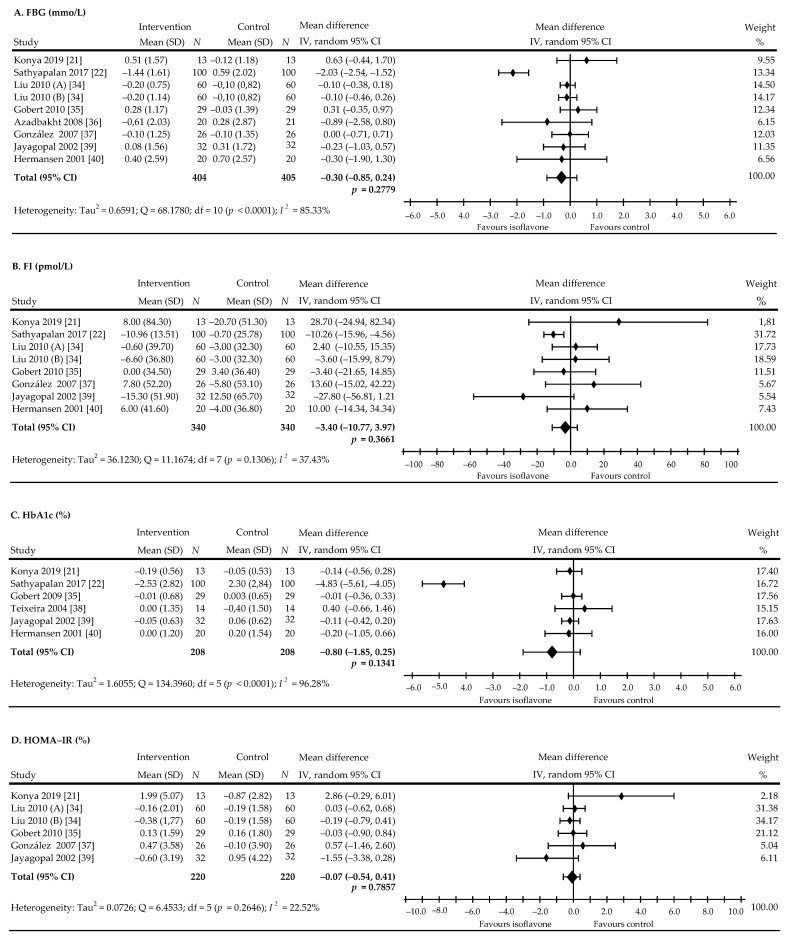
Forest plots showing the difference in glycemic control in all trials between soy isoflavone—administered and control groups. (**A**): FBG, (**B**): FI, (**C**): HbA1c, (**D**): HOMA-IR. Data calculated from the random-effects model are presented as weighted mean difference and 95% CI. The horizontal lines denote the 95% CIs, some of which extend beyond the limits of the scales. Letter in parentheses following the author’s name indicate a study with more than one treatment arm.

The present meta-analysis examined the effect of soy protein isoflavones on glycemic control. Eight trials with nine comparisons involving 721 patients (360 in the treated group and 361 in the control group) studied the effect of soy isoflavones on FBG. In five comparisons, as compared with control, a non-significant decrease in FBG was shown, but the reduction was statistically significant in one [22], while two trials noticed non-significant increase of values, and in one trial, no changes were observed. In turn, eight comparisons from seven trials based on data from 680 subjects (treated—340; control—340) analyzed the effect of isoflavones on FI levels. A non-significant decrease in the FI level was recorded in 4 comparisons, in one of these, the decrease was significant [22] while a non-significant increase in the FI was recorded in four comparisons. Moreover, six studies evaluated the effect of isoflavones on HbA1c in 416 people with T2DM (treated—208; control—200). Here, a decrease of HbA1c values was found in five studies, including one study where a significant reduction was observed [22] and 1 wherein a non-significant increase in value was assessed. Five studies, including 380 subjects (treated—220; control—160), were assigned to assessing the impact of isoflavones on the level of the HOMA-IR. In these, a non-significant decrease in HOMA-IR indicator values were seen in three and an increase was noted in three comparisons. The overall pooled net effect of soy isoflavones supplementation on glycemic metabolism was ‒0.30 mmol/L (95% CI, ‒0.85 to 0.24), *p* = 0.2779, this was accompanied by high heterogeneity: I^2^ = 85.33% for FBG (Figure 4A); ‒3.40 mmol/L (95% CI, ‒10.77 to 3.97), *p* = 0.3661, I^2^ = 37.43% for FI (Figure 4B); ‒0.80% (95% CI, ‒1.85 to 0.25), *p* = 0.1341, with notice of high heterogeneity: I^2^ = 96.28% for HbA1c (Figure 4C); and ‒0.07% (95% CI, ‒0.54 to 0.41), *p* = 0.7857, I2 = 22.52% for HOMA-IR (Figure 4D).

Publication bias was examined by analyzing a series of regression tests for all pooled effects. Begg and Mazumdar’s test for rank correlation indicated evidence of publication bias: Kendall’s tau = ‒0.7333, z = ‒2.0665, *p* = 0.0388. In turn, Egger’s test revealed no evidence of publication bias: intercept = ‒0.1931, t = ‒0.1032, *p* = 0.9207. Simultaneously, the results of Begg and Mazumdar’s test for rank correlation did not indicate publication bias in the meta-analysis of other components of glucose metabolism: Kendall’s tau = 0.3333, z = 1.0513, *p* = 0.2931 for insulin and Kendall’s tau = 0.4667, z = 1.3151, *p* = 0.1885 for HOMA-IR. The analysis did not generate results for Begg and Mazumdar’s test. Moreover, the results of Egger’s test for regression showed no evidence of publication bias for insulin, HbA1c and HOMA-IR: intercept: 1.2126, t = 1.8271, *p* = 0.1175; intercept: ‒5.0560, t = ‒0.9855, *p* = 0.3802; and 0.6407, t = 0.6994, *p* = 0.5632; respectively. 

To explore the possible influence of covariates on net glycemic change, a subgroup analysis was additionally conducted on the basis of eight pre-specified factors (study design, follow-up period, age, BMI, diabetes duration, isoflavone doses, diabetes therapy, and diabetes complications) as presented in Table 2.

The results of subgroups analysis showed no statistically significant differences between groups for HOMA-IR. However, soy isoflavones supplementation in subjects’ age ≤60 statistically significantly reduced HbA1c levels (*p* < 0.0001). Moreover, diabetes duration more than 5 years statistically significantly reduced FBG and FI levels (*p* = 0.0003 for FBG, and *p* = 0.0004 for FI; respectively), and additionally the levels of FBG and FI were decreased when diabetes with complications occurred (*p* = 0.0003 for FBG, and *p* = 0.0004 for FI; respectively).

The multivariate meta-regression analysis suggested that included covariates had no significant influence on FI and HOMA-IR. However, the diabetes duration and complications variables were excluded from the analysis for HOMA-IR due to the occurrence in only one group. Simultaneously, multivariate meta-regression showed that most covariates had no significant effect on FBG, except for the duration of diabetes (β = ‒1.400, *p* = 0.001). Subject age (β = ‒2.297, *p* < 0.001) and diabetes duration (β = ‒1.857, *p* = 0.007) had significant influence on HbA1c (Supplementary Table S1).

### 3.4. The Effect of Soy Isoflavones on Lipid Levels in Patients with Type 2 Diabetes

The levels of individual components of the lipid profile were analyzed in 10 RCTs before and after administration of soy protein and/or isoflavones [21,22,23,36,37,38,39,40,41,42]. In total, 615 subjects participated in the study, including 307 in the active groups and 308 in the control groups. In comparison with the control group, total cholesterol decreased in eight studies, but the decrease was statistically significant only in one [22], while one showed a slight increase in level [37]. The concentration of LDL-C decreased in six studies [21,36,39,40,41,42] and in three studies this was statistically significant [22,23,34], while an insignificant increase was observed in one study [37]. Furthermore, five studies showed a non-significant decrease in HDL-C [21,22,36,39,41], one study showed no changes [37] and four studies showed no significant increase in the level [23,38,40,42]. TAG values decreased in four studies [36,39,40,42] and the reduction was significant in two studies [22,23], no changes were observed in one study [37] and a non-significant increase was noted in three studies [21,38,41].

**Figure 5:**
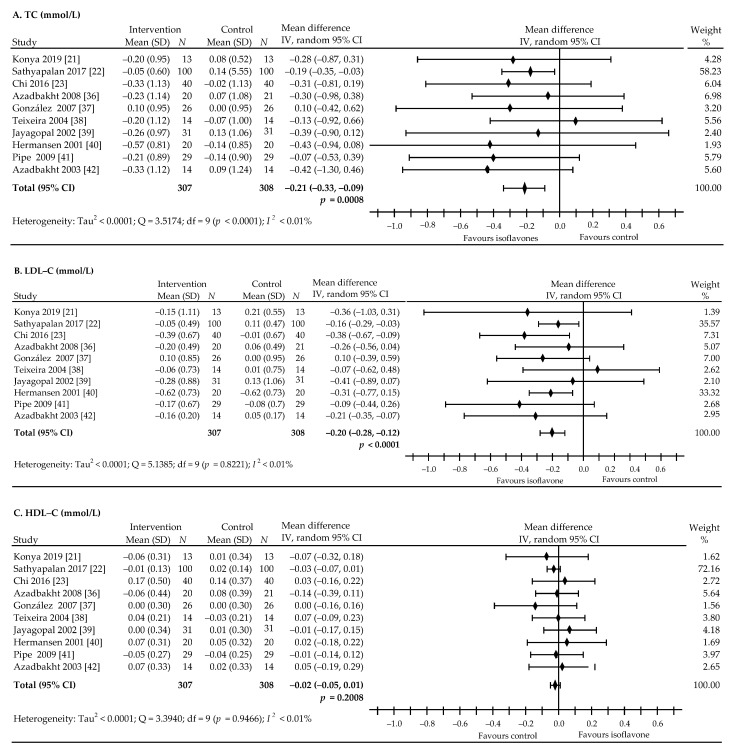

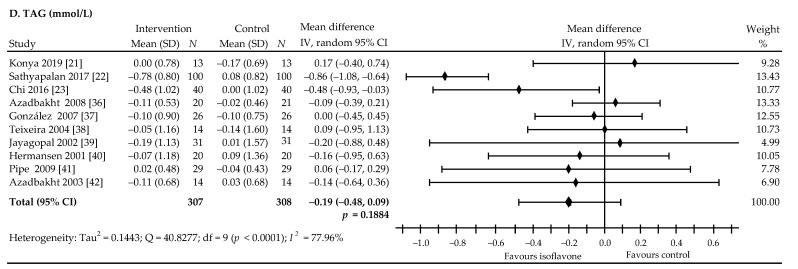
Forest plots showing the difference in lipid-profile components in all trials between soy isoflavone-administered and control groups. (**A**): TC, (**B**): LDL-C, (**C**): HDL-C, (**D**): TAG. Data calculated from the random-effects model are presented as weighted mean difference and 95% CI. The horizontal lines denote the 95% CIs, some of which extend beyond the limits of the scales.

The pooled estimate revealed that the intake of soy isoflavones was associated with statistically significance decreases in plasma concentrations of TC: ‒0.21 mmol/L (95% CI, ‒0.33 to ‒0.09 mmol/L), *p* = 0.0008, I^2^ < 0.01% (Figure 5A) and LDL-C: ‒0.20 mmol/L (95% CI, ‒0.28 to ‒0.12 mmol/L), *p* < 0.0001; I^2^ < 0.01% (Figure 5B). However, isoflavone preparations had no significant effects on the plasma levels of HDL-C: ‒0.02 mmol/L (95% CI: ‒0.05 to 0.01 mmol/L), *p* = 0.2008, I^2^ < 0.01% (Figure 5C) and TAG: ‒0.19 mmol/L (95% CI, ‒0.48 to 0.09 mmol/L), *p* = 0.1884, I^2^ = 77.96% (Figure 5D), compared to the control. Taking into account the possible confounding factor, i.e., a higher dose (435 mg) in the study by Chi et al. [23], additional analysis was performed on the effect of isoflavones on the lipid profile after excluding the extreme value of 435 mg. However, we found that after exclusion, the results did not affect the final outcome of the presented analysis: TC: ‒0.20 (95% CI, ‒0.33 to ‒0.08 mmol/L), *p* = 0.0016, LDL-C: ‒0.19 (95% CI, ‒0.27 to ‒0.10 mmol/L), *p* < 0.0001, HDL-C: ‒0.02 (95% CI, ‒0.05, 0.01 mmol/L), *p* = 0.1777, TAG: ‒0.15 (95% CI, ‒0.47 to 0.16 mmol/L), *p* = 0.3362.

Begg’s and Mazumdar rank correlation test indicated no evidence of publication bias for TC (Kendall’s tau = ‒0.1556; z = ‒0.6261; *p* = 0.53), LDL-C (Kendall’s tau = ‒0.0667; z = ‒0.2683, *p* = 0.7884), HDL-C (Kendall’s tau = ‒0.0222, z = ‒0.0894, *p* = 0.93) and TAG (Kendall’s tau = ‒0.1429, z = ‒0.4949, *p* = 0.62). Moreover, the results of Egger’s test for regression revealed no evidence of publication bias for TC, LDL-C, HDL-C and TAG: ‒0.2049, t = ‒0.4284, *p* = 0.68; intercept: ‒0.25, t = ‒0.4284, *p* = 0.69; intercept: 0.3289, t = 1.1212, *p* = 0.29; and 1.4968, t = 0.9616, *p* = 0.36; respectively. 

To investigate the possible effect of covariates on lipid profile alteration, a subgroup analysis was additionally performed taking into account participant characteristics that included age, BMI and diabetes duration, study design, follow-up period, isoflavones dosing, diabetes therapy and diabetes complications (Table 3). The results of subgroups analysis presented non-significance between group differences for HDL-C. Here, the statistically significant effect of soy isoflavones on changes in TAG was only observed in the subgroup of people aged ≤60 years (*p* = 0.0001). However, diet and/or drug administration statistically were observed to significantly reduce TC (*p* = 0.0028) and LDL-C levels (*p* = 0.0014). In addition, levels of TC were decreased among patients in parallel group studies (*p* = 0.0041), in obese patients (*p* = 0.0031), and in patients that used higher doses of isoflavones (*p* = 0.0022).

Furthermore, multivariate meta-regression analysis suggested that the included covariates had no significant influence on HDL-C and TAG. Concurrently, multivariate meta-regression showed that most covariates had no significant effect on TC, except for the follow-up time (β = 0.242, *p* = 0.035). However, follow-time (β = 0.202, *p* = 0.014), BMI (β = ‒0.999, *p* = 0.022), diabetes duration (β = 0.978, *p* = 0.008), and isoflavone intake (β = ‒0.483, *p* = 0.021) were found to have significant influence on LDL-C (Supplementary Table S2).

## 4. Discussion

Our meta-analysis demonstrated a significant reduction in the concentration of TC (‒0.21 mmol/L, *p* = 0.0008) and LDL-C (‒0.20 mmol/L, *p* < 0.0001) in the plasma, while the levels of HDL-C (‒0.02 mmol/L, *p* = 0.2008) and TAG (‒0.19 mmol/L, *p* = 0.1884) did not change significantly after ingesting soy isoflavone supplements. Zhang et al. [43] also showed significant reduction in TC (‒0.39, *p* < 0.01) and in LDL-C (‒0.30, *p* < 0.01) and non-significant decrease in HDL-C (‒0.05, *p* = 0.55) and TAG (‒0.094, *p* = 0.27), while Yang et al. [44] noted significant reduction in TC (‒0.42, *p* < 0.05), TAG (‒0.22, *p* < 0.05), significant reduction in LDL-C (‒0.30, *p* = 0.05) and significant increase in HDL-C (0.05, *p* < 0.05). Furthermore, Soltanipour et al. [45] observed significant reduction of TC (‒0.47, *p* < 0.01). In addition, soy products consumption was seen to be beneficial in decreasing LDL-C and TAG, but had no significant effects on HDL-C (results not shown). Beyond the aforementioned, Giordano et al. revealed in their study that soy isoflavones increased plasma TC concentrations and decreased triglyceride ones—adding further evidence to the notion that soy isoflavones have assorted effects on cardiometabolic risk factors [46].

Simultaneously our meta-analysis for the effects on glycemic control revealed that soy protein and/or isoflavones are not significantly effective in reducing circulating glucose levels. In addition, the outcome of our meta-analysis of randomized controlled trials has indicated that soy protein and/or isoflavones supplementation has no statistical significance effect on glycemic control in T2DM (FBG: ‒0.30 mmol/L, *p* = 0.28; FI: ‒3.40 pmol/L, *p* = 0.37; HbA1c: ‒0.80%, *p* = 0.13; and HOMA-IR: ‒0.07, *p* = 0.79). These results are similar to those of Yang et al. [44], who, in 2011, also did not show any significant effect of soy protein and/or isoflavones on the level of FBG, FI and HbA1c. In turn, the meta-analysis by Zhang et al. [43], published in 2016 and based on eight trials with 13 comparisons revealed significant changes in the FBG, FI and HOMA-IR values after administering soy preparations (‒0.207, *p* = 0.015; ‒0.29, *p* = 0.01; and ‒0.346, *p* < 0.01; respectively). Moreover, a recently published meta-analysis by Soltanipour et al. [45] reported that, according to the data from 14 RCTs, soy consumption had significant effects on HOMA-IR level (‒0.25, *p* < 0.01), in the absence of significant effects on FBG (‒0.14, *p* = 0.09; FI: ‒0.11, *p* = 0.11; and HbA1c: ‒0.22, *p* = 0.18). 

The observed differences in outcomes between earlier meta-analyses and our study can be result of differences in the inclusion criteria. We relied only on studies assessing the effects of isoflavones contained in soy protein or on the isoflavones alone. Yang et al. [44] used a study by Anderson et al. [47] in which only soy protein was used. In turn, the meta-analysis by Zhang et al. [43] included research with soy protein alone [47] or black soybean peptides [48], but also a study involving nondiabetic people with metabolic syndrome [49]. Furthermore, Soltanipour et al. [45], in addition to including seven out of 16 studies using isolated soy protein and isoflavones [23,35,36,37,38,39,40,42], also analyzed studies using different types of soy products such as soy milk [50,51], bread fortified with soy flour [52], soy germ pasta enriched in isoflavones [20], multifilament soy protein-based diabetes-specific food [17], as well as other preparations containing native starch banana [53] or flavan-3-ols/isoflavones [54].

The molecular and physiological mechanisms underlying the metabolic action of phytoestrogens components containing in soybean have not yet been fully recognized. The studies conducted with soy dietary isoflavones and isoflavone alone in cell culture or in animal models and human studies have definitely demonstrated that isoflavones can improve some parameters associated with the course of diabetes. In addition, the structural similarity between soy isoflavones and endogenous 17-β-estradiol suggests that isoflavones, by binding to estrogen receptors (ERs), lead to gene activation and beneficial effects on glucose and lipid metabolism [55,56]. 

There is some evidence to intimate that estrogen receptor (ER) binding is only part of the isoflavone effect [57]. Genistein and daidzein (and its metabolite equol), improve glycemic control, and significantly alter glucose homeostasis through: (a) stimulating insulin secretion by inhibiting tyrosine kinase (TK) [58,59]; (b) activating adenosine 5′-monophosphate (AMP)-activated protein kinase (AMPK)—which results in decrease blood glucose in the liver, while stimulating glucose uptake independently of insulin in skeletal muscles and modulating glucose transport in peripheral tissue [60]; (c) activating the peroxisome proliferator-activated receptor gamma (PPARγ); thus, enhancing the expression and translocation of GLUT-1 and GLUT-4—which results in increased glucose uptake in adipocytes and muscle cells and subsequent reduction in plasma glucose levels [61]; (d) inhibiting alpha-glucosidase (AG)—which leads to slowing down the absorption of glucose in the gut [62]; and (e) directly modulating pancreatic beta-cell function and conferring protection against apoptosis through mechanisms that involve cyclic AMP/Protein Kinase A (cAMP/PKA) signaling [63,64].

Moreover, isoflavones can also regulate lipid metabolism without the mediation of estrogen receptors; increase expression of PPARα and activate AMPK—which results in increased activity of genes involved in lipoprotein metabolism; reduce TG-rich particle production and increase their lipolysis; promote HDL metabolism and promote the uptake, utilization and catabolism of fatty acid [65,66,67]. Furthermore, isoflavones can inhibit the expression and activity of the sterol regulatory element binding protein-1c (SREBP-1c) and carbohydrate regulatory element binding protein-1 (ChREBP)—proteins that enhance the expression of lipogenic genes and key enzymes involved in de novo lipogenesis [68,69]. Other possible mechanisms of soy isoflavones that may modulate lipoprotein metabolism, include their effects on several enzymes important in lipid transformation, including lipoprotein lipase (LPL), hepatic lipase (HL) also called hepatic triglyceride lipase (HTGL), and 7alpha-hydroxylase [70,71]. 

### Limitations of This Study

Limitations of the presented meta-analysis must be acknowledged. First, the pooled population analyzed in our meta-analysis included a limited number of subjects because the sample size in some of the clinical trials was small. Secondly, the duration of treatment in some studies was short (<2 months), which could reduce the effect of soy isoflavones supplementation. Thirdly, the selected studies used different doses and different forms of soy isoflavones (methylated forms, glycosides, and aglycones)—which could have affected the results. Fourthly, the clinical effectiveness of soy isoflavones may be limited by the ability to transform soy isoflavones to the more potent estrogenic metabolite (equol). High variability in equol production is attributable to interindividual differences in the composition of the intestinal microflora; only approximately one-third to one-half of the population is able to metabolize daidzein to equol [55,56,72].

## 5. Conclusions

Our analysis found that consumption of soy isoflavones brought about a statistically significant reduction in total and LDL cholesterol, while simultaneously demonstrating no significant effects on HDL and TAG. Influence of soy isoflavones on glucose levels has been shown to be statistically insignificant. Moreover, the ability of both extracted isoflavone and soy protein with isoflavones to modulate the lipid profile suggests benefits in preventing cardiovascular events in people with type 2 diabetes. However, further multicenter studies based on a larger pool of research material and a well accurately defined dose of isoflavones are necessary to determine their beneficial effects on glucose and lipid metabolism.

## Figures and Tables

**Table 1:** Characteristics of selected randomized clinical studies assessing effect of soy isoflavones on glycemic control and lipid profile in type 2 diabetes.

First Author	Study Design	Study Population	Intervention (Daily Dose)	Control (Daily Dose)	Dietary Advice during Study	Outcome Measures
Data [Reference]		(DM Duration; Diabetes Therapy)				
Country	Trial Duration	“Conditions Accompany Diabetes”				
Konya 2019 [21] Qatar	Parallel groups; 2-w run-in, 8-w follow-up.	*n* = 26, 8 women and 18 men; age 65.1 ± 7.3 y; BMI 30.5 ± 5.1 [4.4 ± 3.7; diet or metformin]	16 g SP, 32 mg ISF; in form of bars	16 g SP alone; in form of bars	Maintained current diet; avoid dietary products with a high-ISF content	TC, LDL-C, HDL-C, TAG FBG, FI, HbA1c, HOMA-IR
Sathyapalan 2017 [22] UK	Parallel groups, 3-mo follow-up.	*n* = 200 men; age 52.0 y †; BMI 31.8 †. [7.3 *; stable drugs for T2DM] “subclinical hypogonadism’	15 g SP, 166 mg ISF; in form of bars	15 g SP alone: in form of bars	Avoiding soy products, nutritional, mineral and vitamin supplements	TC, LDL-C, HDL-C, TAG FBG, FI, HbA1c
Chi 2016 [23] China	Parallel groups, 2-mo follow-up.	*n* = 80 women; age 51.9 ± 11.0 y; BMI 24.1 ± 0. 8[N/A; N/A]	435 mg IAE (52.2% Gen, 47.8% Dai) capsule	Starch; capsule	ISF intake from foods was restricted to less than 19 mg/day	TC, LDL-C, HDL-C, TAG
Liu 2010 [34] Hong Kong	Three-arm study, parallel groups; 2-w run-in, 24-w follow-up.	*n* = 180 women; age 56.1 ± 4.3 y; BMI 24.5 ± 3.7 [untreated early diabetes]	A. 15 g SP, 100 g ISF (≈25 mg Agl). B. 15 g MP, 100 mg ISF (≈25 mg Agl); powder	15 g MP; powder	Maintained habitual diet; not to take supplements containing isoflavones or other extracts	FBG, FI, HOMA-IR
Gobert 2010 [35]	Cross-over trial; 4-w washout, 57-d active phase.	*n* = 29, 13 women and 16 men; age 60.1 ± 9.6 y; BMI 29.6 ± 4.1	40 g SP; 88 mg IAE (65% Gen, 31% Dai, 4% Gly powder)	40 g MP powder	Maintained habitual diet; other phytoestrogen	FBG, FI, HbA1c, HOMA-IR
Azadbakht 2008 [36] Iran	Parallel groups *; 4-y follow-up.	*n* = 41, 23 women and 18 men; age 62.1 ± 12.1 y; 71.5 ± 8.5 kg # [10.0 ± 3.0; insulin or oral drugs] “nephropathy”	16 ± 9 g SP, ≈43 ISF ‡	≈20 g AP §	Maintained current diet	TC, LDL-C, HDL-C, TAG FBG
González 2007 [37] UK	Cross-over trial; 4-w washout, 12-w active phase.	*n* = 26 women; age N/A; BMI 30.8 ± 5.9 [N/A; non medications]	132 mg IAE (35% Gen, 37% Dai, 10% Gly); pill	Microcrystalline cellulose, pill	Diet-controlled diabetes	TC, LDL-C, HDL-C, TAG FBG, FI, HOMA-IR
Teixeira 2004 [38] Portugal	Cross-over trial *; 4-w washout, 8-w active phase.	*n* = 14 men; age 53–73 y; BMI 29.8 ± 2.3; [~14.0 y; insulin] “early stages nephropathy”	0.5 g/kg SP isolate, 2.0 mg/g protein IAE, powder	0.5 g/kg casein; powder	Diet excluding foods containing soy	TC, LDL-C, HDL-C, TAG HbA1C
Jayagopal 2002 [39] UK	Cross-over trial; 2-w washout, 12-w active phase.	*n* = 32 women; age 63.5 ± 12.1 y; BMI 32.2 ± 5.0 [2.6 ± 2.7; non medications]	30 g SP isolate, 135 mg ISF (95% glucosides)	Microcrystalline cellulose 30 g	Recommended maintain a diabetes diet	TC, LDL-C, HDL-C, TAG FBG, FI, HbA1c, HOMA-IR
Hermansen 2001 [40] Norway	Cross-over trial; 3-w washout, 6-w active phase.	*n* = 20. 6 women and 14 men; age 63.6 ± 7.5 y; BMI 30.2 ± 4.2 [3.0 ± 2.7; oral drugs]	50 g SP isolate, ≥165 mg ISF; 20 g cotyledon fiber	50 g casein; 20 g cellulose	Diet set and controlled by a dietitians	TC, LDL-C, HDL-C, TAG FBG, FI, HbA1C
Pipe 2009 [41] Canada	Cross-over trial; 4-w washout, 57-d active phase.	*n* = 29, 13 women and 16 men; age 60.1 ± 9.6 y; BMI 29.6 ± 4.1	40 g SP; 88 mg IAE (65% Gen, 31% Dai, 4% Gly powder)	40 g MP powder	intake were prohibited	TC, LDL-C, HDL-C, TAG
Azadbakht 2003 [42] Iran	Crossover trial *; 4-w washout, 7-w active phase.	*n* = 14, 4 women and 10 men; age 62.5 ± 12.1 y; 70.6 ± 10.3 kg # [N/A; N/A], ‘nephropathy’	≈20 g SP, ≈43 mg ISF ‡	≈20 g AP §	The alternate test diet	TC, LDL-C, HDL-C, TAG

Data are presented as mean ± standard deviation; range or mean; * non-blinded design; † values are provided as medians; ‡ 0.8 g protein/kg (35% textured soy protein, 35% animal protein, 30% vegetable protein; § 0.8 g protein/kg (70% animal protein, 30% vegetable protein); # baseline body weight values are only reported when no data on BMI were available.

**Table 2:** Pooled estimates of treatment effect on glycemic control in subgroups of trials.

	FBG (mmol/L)				FI (pmol/L)					HbA1c (%)					HOMA-IR (%)					
Variables	n	N	WMD (95% CI)	*p*	I^2^ (%)	n	N	WMD (95% CI)	*p*	I^2^ (%)	n	N	WMD (95% CI)	*p*	I^2^ (%)	n	N	WMD (95% CI)	*p*	I^2^ (%)
**Overall effects**	9	721	−0.30 (−0.85, 0.24)	0.2279	85.66	8	680	−3.40 (−10.77, 3.97)	0.3661	37.43	6	416	−0.80 (−1.85, 0.25)	0.1341	96.25	6	440	−0.07 (−0.54, 0.41)	0.7857	22.52
**Study design**																				
Parallel groups	5	507	−0.50 (−1.34, 0.33)	0.2379	92.12	4	466	−4.54 (−12.83, 3.75)	0.2831	42.98	2	226	−2.47 (−7.07, 2.12)	0.2916	99.06	3	266	0.04 (−0.63, 0.72)	0.8977	43.73
Cross-over	4	214	0.04 (−0.36, 0.44)	0.8527	<0.01	4	214	−1.46 (−17.47, 14.55)	0.8585	41.47	4	190	−0.06 (−0.27, 0.16)	0.6126	<0.01	3	174	−0.26 (−1.25, 0.73)	0.6048	20.04
**Follow-up period**																				
≤8 weeks	3	124	0.32 (−0.21, 0.85)	0.2342	<0.01	3	124	3.31 (−10.78, 17.40)	0.6457	<0.01	4	152	−0.05 (−0.30, 0.20)	0.6919	<0.01	2	84	1.00 (−1.71, 3.72)	0.4695	66.62
>8 weeks	6	597	−0.53 (−1.21, 0.14)	0.1222	89.71	5	556	−5.33 (−13.81, 3.15)	0.2176	46.62	2	264	−2.46 (−7.08, 2.17)	0.2981	99.17	4	356	−0.14 (−0.56, 0.29)	0.5249	1.08
**Age**																				
≤60 years	3	440	−0.72 (−1.75, 0.31)	0.1699	95.74	3	440	−5.65 (−13.23, 1.92)	0.1434	43.26	1	200	−4.83 (−5.61, −4.05)	<0.0001	N/R	2	240	−0.09 (−0.53, 0.35)	0.6944	<0.01
>60 years	5	229	0.09 (−0.34, 0.52)	0.6879	<0.01	4	188	−2.22 (−20.43, 5.99)	0.8112	42.78	5	216	−0.07 (−0.27, 0.12)	0.4533	<0.01	3	148	0.03 (−1.74, 1.79)	0.9763	65.74
**Body mass index**																				
<30 kg/m^2^	4	339	0.07 (−0.28, 0.14)	0.4989	<0.01	3	298	−1.25 (−9.29, 6.79)	0.7606	<0.01	2	86	0.03 (−0.30, 0.35)	0.8742	<0.01	3	298	−0.08 (−0.47, 0.32)	0.7031	<0.01
≥30 kg/m^2^	5	382	−0.43 (−1.54, 0.69)	0.4511	89.33	5	382	−2.46 (−17.58, 12.66)	0.7494	52.49	4	330	−1.30 (−2.96, 0.37)	0.1269	97.63	3	142	0.36 (−2.42, 3.14)	0.1669	37.01
**Diabetes** **duration** *																				
<5 years	6	428	−0.05 (−0.25, 0.15)	0.6238	<0.01	6	428	−1.41 (−9.58, 6.76)	0.7347	12.76	4	188	−0.09 (−0.28, 0.11)	0.3682	<0.01	5	388	−0.10 (−0.63, 0.43)	0.4104	33.93
≥5 years	2	241	−1.76 (−2.71, −0.81)	0.0003	37.46	1	200	−10.26 (−15.96, −4.56)	0.0004	N/R	2	228	−2.23 (−7.35, 2.90)	0.3945	98.35					
**Isoflavone intake**																				
<100 mg/d	3	125	0.26 (−0.34, 0.85)	0.3961	11.29	4	324	−0.59 (−8.54, 7.36)	0.8839	<0.01	3	112	−0.04 (−0.29, 0.22)	0.7820	<0.01	2	84	1.00 (−1.71, 3.72)	0.4695	66.62
≥100 mg/d	6	596	−0.48 (−1.15, 0.19)	0.1618	89.63	4	356	−4.85 (−19.79, 10.10)	0.5249	54.10	3	304	−1.70 (−4.56, 1.15)	00.2426	98.36	4	356	−0.14 (−0.56, 0.29)	0.5249	1.08
**Diabetes therapy**																				
Non medications	5	414	−0.06 (−0.26, 0.13)	0.5159	<0.01	5	414	−2.17 (−10.59, 6.24)	0.6127	15.07	2	122	−0.07 (−0.30, 0.16)	0.5658	<0.01	5	414	−0.12 (−0.49, 0.26)	0.5446	<0.01
Diet and/or drugs	4	307	−0.69 (−2.19, 0.81)	0.3651	86.36	3	266	0.32 (−19.22, 19.87)	0.9741	54.76	4	294	−1.20 (−3.49, 1.09)	0.3052	97.45	1	26	2.86 (−0.29, 6.01)	0.0755	
**Complications**																				
without	7	480	−0.05 (−0.24, 0.14)	0.6366	<0.01	7	480	−0.36 (−8.15, 7.44)	0.9287	10.68	4	188	−0.09 (−0.28, 0.11)	0.3685	<0.01	6	440	−0.07 (−0.54, 0.41)	0.7857	22.52
with	2	241	−1.76 (−2.71, −0.80)	0.0003	37.67	1	200	−10.26 (−15.96, −4.56)	0.0004	98.35	2	228	−2.23 (−7.35, 2.90)	0.3945	98.35					

HbA1c—glycated hemoglobin; CI—confidence interval; HOMA-IR—homeostatic model assessment of insulin resistance; I2—coefficient of inconsistency; n—number of comparisons; N—sample size; *p*—probability value; N/R—not reported; WMD—weighted mean difference; *—data not available in trial of González et al. 2007 [37]—study not included in the HbA1c analysis

**Table 3:** Pooled estimates of treatment effect on lipid profile in subgroups of trials.

	TC (mmol/L)					LDL-C (mmol/L)					HDL-C (mmol/L)					TAG (mmol/L)				
Variables	*n*	N	WMD (95% CI)	*p*	I^2^ (%)	*n*	N	WMD (95% CI)	*p*	I^2^ (%)	*n*	N	WMD (95% CI)	*p*	I^2^ (%)	*n*	N	WMD (95% CI)	*p*	I^2^ (%)
Overall effects	10	615	−0.21 (−0.33, −0.09)	0.0008	<0.01	10	615	−0.20 (−0.28, −0.120	<0.0001	<0.01	10	615	−0.02 (−0.05, 0.01)	0.2008	<0.01	10	615	0.19 (−0.48, 0.09)	0.1884	77.96
Study design																				
parallel group	4	347	-0.21 (−0.35, −0.07)	0.0041	<0.01	4	347	−0.21 (−0.32, −0.10)	0.0002	<0.01	4	347	−0.03 (−0.07, 0.01)	0.0922	<0.01	4	347	−0.34 (−0.83, 0.14)	0.1620	86.50
cross-over	6	268	−0.20 (−0.43, 0.03)	0.0828	<0.01	6	268	−0.19 (−0.31, −0.08)	0.0009	<0.01	6	268	0.02 (−0.05, 0.08)	0.6564	<0.01	6	268	−0.00 (−0.18, 0.18)	0.9816	<0.01
Follow-up period																				
≤8 weeks	6	260	−0.26 (−0.49, −0.03)	0.0287	<0.01	6	260	−0.23 (−0.34, −0.12)	0.0001	<0.01	6	260	0.02 (−0.06, 0.09)	0.6238	<0.01	6	260	−0.05 (−0.24, 0.13)	0.5689	5.07
>8 weeks	4	355	−0.19 (−0.33, −0.05)	0.0095	<0.01	4	355	−0.17 (−0.29, −0.06)	0.0029	<0.01	4	355	−0.03 (−0.06, 0.01)	0.0988	<0.01	4	350	−0.31 (−0.81, 0.19)	0.2875	86.55
Age *																				
≤60 years	2	280	−0.20 (−0.35, −0.05)	0.0094	<0.01	2	280	−0.23 (−0.43, −0.03)	0.0248	40.10	2	280	−0.03 (−0.06, 0.01)	0.1380	<0.01	2	280	−0.72 (−1.08, −0.36)	0.0001	54.65
>60 years	7	283	−0.28 (−0.50, −0.06)	0.0134	<0.01	7	283	−0.22 (−0.34, −0.11)	0.0001	<0.01	7	283	0.00 (−0.07, 0.07)	0.9924	<0.01	7	283	−0.01 (−0.17, 0.14)	0.8772	<0.01
Body mass index †																				
<30 kg/m^2^	3	166	−0.17 (−0.48, 0.14)	0.2730	<0.01	3	166	−0.23 (−0.44, −0.02)	0.0283	<0.01	3	166	0.03 (−0.06, 0.12)	0.5802	<0.01	3	166	−0.13 (−0.54, 0.28)	0.5338	55.42
≥30 kg/m^2^	5	380	−0.21 (−0.34, −0.07)	0.0031	<0.01	5	380	−0.18 (−0.29, −0.06)	0.0035	<0.01	5	380	−0.03 (−0.06, 0.01)	0.1328	<0.01	5	380	−0.24 (−0.74, 0.26)	0.3456	81.16
Diabetes duration ‡																				
<5 years	4	186	−0.28 (−0.54, −0.02)	0.0316	<0.01	4	186	−0.25 (−0.47, −0.02)	0.0349	<0.01	4	186	−0.01 (−0.10, 0.07)	0.8012	<0.01	4	186	0.04 (−0.16, 0.24)	0.7179	<0.01
≥5 years	3	269	−0.19 (−0.35, −0.04)	0.0129	<0.01	3	269	−0.17 (−0.29, −0.05)	0.0047	<0.01	3	269	−0.02 (−0.08, 0.03)	0.4296	12.00	3	269	−0.36 (−1.02, 0.29)	0.2753	88.59
Isoflavone intake																				
<100 mg/d	4	152	−0.21 (−0.51, 0.09)	0.1690	<0.01	4	152	−0.21 (−0.32, −0.09)	0.0004	<0.01	4	152	−0.03 (−0.13, 0.07)	0.5648	<0.01	4	152	−0.14 (−0.64, 0.36)	0.5860	<0.01
≥100 mg/d	6	463	−0.21 (−0.34, −0.07)	0.0022	<0.01	6	463	−0.19 (−0.30, −0.09)	0.0004	<0.01	6	463	0.01 (−0.04, 0.06)	0.2481	<0.01	6	463	−0.35 (−0.73, 0.03)	0.0727	69.56
Diabetes therapy #																				
non medications	3	172	−0.12 (−0.40, 0.16)	0.4085	<0.01	3	172	−0.13 (−0.38, 0.13)	0.3400	7.72	3	172	−0.01 (−0.11, 0.10)	0.9093	<0.01	3	172	0.03 (−0.17, 0.23)	0.7971	<0.01
diet and/or drugs	5	355	−0.22 (−0.36, −0.07)	0.0028	<0.01	5	355	−0.19 (−0.30, −0.07)	0.0014	<0.01	5	355	−0.03 (−0.06, 0.01)	0.1439	<0.01	5	355	−0.23 (−0.73, 0.28)	0.3793	83.47
Complications																				
without	6	318	−0.22 (−0.43, −0.02)	0.0347	<0.01	6	318	−0.25 (−0.42, −0.08)	0.0039	<0.01	6	318	−0.00 (−0.07, 0.07)	0.9233	<0.01	6	318	−0.05 (−0.23, 0.13)	0.5875	5.68
with §	4	297	−0.20 (−0.35, −0.05)	0.0090	<0.01	4	297	−0.19 (−0.28, −0.10)	0.0000	<0.01	4	297	−0.03 (−0.06, 0.01)	0.1660	<0.01	4	297	−0.32 (−0.84, 0.20)	0.2343	85.00

Abbreviations: CI—confidence interval; I2—coefficient of inconsistency; n—number of comparisons; N—number of participants; P—probability value; WMD—weighted mean difference *—data not available in trial of González et al. 2007 [37]. †—data not available in trials of Azadbakht et al. 2008 [36] and Azadbakht et al. 2003 [42] ‡—data not available in trials of Chi et al. 2016 [33], González et al. 2007 [37] and Azadbakht et al. 2003 [42] #—data not available in trials of Chi et al. 2016 [33] and Azadbakht et al. 2003 [42] §—nephropathy; obesity; hypertension and proteinuria; subclinical hypogonadism.

## Data Availability

Not applicable.

## Supplementary Materials

The following are available online at https://www.mdpi.com/article/10.3390/nu13061886/s1, Table S1: The results of multivariate meta-regression- Glucose. Table S2: The results of multivariate meta-regression—Lipids.
