# Supplementary material for: Effects of Soy Isoflavones on Glycemic Control and Lipid Profile in Patients with Type 2 Diabetes: A Systematic Review and Meta-Analysis of Randomized Controlled Trials
Source: Nutrients. 2021 May 31;13(6):1886. doi: 10.3390/nu13061886 (PMC8229139; doi:10.3390/nu13061886)
Supplement: Supplementary file 1 [file nutrients-13-01886-s001.zip › nutrients-1193384-supplementary.pdf]

Supplementary Table S1. The results of multivariate meta-regression- Glucose

Covariate	FG (mmol/L)		FI (pmol/L)		HbA1c (%)		HOMA-IR (%)	
	β (95% CI)	p	β (95% CI)	p	β (95% CI)	p	β (95% CI)	p
**Study design**<br>**parallel group**<br>**cross-over**	0.390 (-0.050, 0.831)	0.256	-10.029 (-40.900, 20.843)	0.677	0.183 (-0.061, 0.426)	0.336	-0.088 (-0.662, 0.487)	0.845
**Follow-up period**<br>**≤8 weeks**<br>**>8 weeks**	-0.631 (-1.128, -0.134)	0.104	-13.271 (-27,181, 0.638)	0.221	-0.043 (-0.242, 0.157)	0.785	-0.551 (-1.187, 0.085)	0.267
**Subjects age***<br>**≤60 years**<br>**>60 years**	-0.509 (-1.143, 0.125)	0.304	-5.519 (-38.654, 27.615)	0.831	-2.297 (-2.756, -1.839)	< 0.001	2.176 (0.186, 4.166)	0.161
**Body mass index†**<br>**<30 kg/m2**<br>**≥30 kg/m2**	0.244 (-0.379, 0.867)	0.615	5.837 (1.290, 10.384)	0.100	1.247 (0.167, 2.327)	0.139	-1.860 (-3.701, -0.019)	0.195
**Diabetes duration‡**<br>**<5 years**<br>**≥5 years**	-1.400 (-1.949, -0.850)	0.001	-15.728 (-27.050, -4.406)	0.075	-1.857 (-2.721, -0.993)	0.006	NA	NA
**Isoflavone intake**<br>**<100 mg /d**<br>**≥100 mg /d**	-0.411 (-1.074, 0.251)	0.426	-11.015 (-22.584, 0.555)	0.222	0.190 (-0.690, 1.070)	0.782	-2.590 (-4.532, -0.648)	0.087
**Diabetes therapy#**<br>**non medications**<br>**diet and/ or drugs**	NA	NA	-18.870 (-33.516, -4.224)	0.099	-0.554 (-1.384, 0.276)	0.392	NA	NA
**Complications**<br>**without**<br>**with§**	NA	NA	NA	NA	NA	NA	NA	NA

Abbreviations: CI, confidence interval; β, the regression coefficient; p, probability value, NA- not available, multivariate meta-regression not generated the results

*, data not available in trial of González et al. 2007 [38].
†, data not available in trials of Azadbakht et al. 2008 [37] and Azadbakht et al. 2003 [40]
‡, data not available in trials of Chi et al. 2016 [33], González et al. 2007 [38] and Azadbakht et al. 2003 [40]
#, data not available in trials of Chi et al. 2016 [33] and Azadbakht et al. 2003 [40]
§, nephropathy; obesity; hypertension and proteinuria; subclinical hypogonadism

Supplementary Table S2. The results of multivariate meta-regression - Lipids

Covariate	TC (mmol/L)		LDL-C (mmol/L)		HDL-C(mmol/L)		TAG (mmol/L)	
	β (95% CI)	p	β (95% CI)	p	β (95% CI)	p	β (95% CI)	p
**Study design parallel group cross-over**	-0.042 (-0.184, 0.100)	0.706	0.085 (-0.020, 0.190)	0.302	-0.008 (-0.055, 0.039)	0.832	0.285 (0.081,0.490)	0.073
**Follow-up period ≤8 weeks >8 weeks**	0.242 (0.095, 0.388)	0.035	0.202 (0.097, 0.307)	0.014	-0.42 (-0.092, 0.009)	0.289	-0.150 (-0.354, 0.054)	0.347
**Subjects age* ≤60 years >60 years**	-0.105 (-0.643; 0.434)	0.803	-0.367 (-0.683, -0.052)	0.136	0.017 (-0.133, 0.166)	0.885	-0.478 (-0.970, 0.014)	0.213
**Body mass index† <30 kg/m2 ≥30 kg/m2**	-0.256 (-0.634; 0.122)	0.385	-0.999 (-1.557, -0.441)	0.022	0.067 (-0.064, 0.198)	0.512	0.248 (-0.049, 0.545)	0.284
**Diabetes duration‡ <5 years ≥5 years**	0.613 (-0.017, 1.243)	0.213	0.978 (0.505, 1.451)	0.008	-0.094 (-0.289, 0.102)	0.540	-0.274 (-0.908, 0.361)	0.580
**Isoflavone intake <100 mg /d ≥100 mg /d**	-0.329 (-0.674, 0.016)	0.221	-0.483 (-0.752, -0.214)	0.021	0.121 (0.002, 0.241)	0.193	0.006 (-0.311, 0.323)	0.981
**Diabetes therapy# non medications diet and/ or drugs**	0.285 (-0.007, 0.576)	0.211	0.345 (0.097, 0.594)	0.075	-0.064 (-0.170, 0.042)	0.438	-0.192 (-0.508, 0.123)	0.435
**Complications without with§**	-0.030 (-0.664, 0.603)	0.951	0.304 (-0.002, 0.610)	0.203	0.004 (-0.204, 0,213)	0.979	0.218 (-0.252, 0.689)	0.552

Abbreviations: CI, confidence interval; β, the regression coefficient; p, probability value

*, data not available in trial of González et al. 2007 [38].

†, data not available in trials of Azadbakht et al. 2008 [37] and Azadbakht et al. 2003 [40]

‡, data not available in trials of Chi et al. 2016 [33], González et al. 2007 [38] and Azadbakht et al. 2003 [40]

#, data not available in trials of Chi et al. 2016 [33] and Azadbakht et al. 2003 [40]

§, nephropathy; obesity; hypertension and proteinuria; subclinical hypogonadism
